# High Pressure Treatment for Improving Water Vapour Barrier Properties of Poly(lactic acid)/Ag Nanocomposite Films

**DOI:** 10.3390/polym10091011

**Published:** 2018-09-11

**Authors:** Hai Chi, Jing Xue, Cheng Zhang, Haiyan Chen, Lin Li, Yuyue Qin

**Affiliations:** 1 Institute of Yunnan Food Safety, Kunming University of Science and Technology, Kunming 650550, China; 18468273140@163.com (H.Chi); 13136640259@163.com (C.Z.); seacome@163.com (H.Che.); 2 State Key Laboratory of Oral Diseases, Sichuan University, Chengdu 610041, China; aqua119@163.com; 3 College of Food Sciences and Engineering, South China University of Technology, Guangzhou 510640, China; felinli@scut.edu.cn

**Keywords:** poly (lactic acid), nano-Ag, high pressure, water vapour barrier property, migration

## Abstract

Effects of high pressure treatment (0, 200 and 400 MPa) on water vapour barrier, microstructure, thermal, and mechanical properties of poly (lactic acid) (PLA)/Ag nanocomposite films were investigated. The migration behavior of nano-Ag from the nanocomposite films in the presence of 50% (*v*/*v*) ethanol as a food simulant was also studied. The water vapour barrier properties increased as pressure was applied to film-forming solutions. High pressure treatment enhanced the mutual effect between PLA and nanoparticles, leading to a more compact network structure in PLA/Ag nanocomposite films. Furthermore, PLA/Ag nanocomposite films treated by high pressure were significantly affected by microstructure, thermal, and mechanical properties when, compared with untreated samples. High pressure treatment at 200 to 400 MPa significantly (*p* < 0.05) reduced the migration of nano-Ag from the films. Overall, high pressure treatment on film-forming solutions showed potential in improving the functional properties of nanocomposite films, especially in relation to water vapour barrier properties.

## 1. Introduction

In recent years, biodegradable polymers represent an alternative to replace petroleum-based resins due to increasing environmental concerns [1,2,3]. Among the different biodegradable materials, poly (lactic acid) (PLA) is widely used for commercial products [4]. PLA is a starch derived from fermented plant originating from renewable resources, such as sugarcane, beetroot, and potato starch [5,6]. It is safe as a food packaging material because it has been approved by the U.S. Food & Drug Administration (FDA) [7].

However, although the hydrophilicity of the PLA-based films are generally good, there is also a poor water vapor barrier properties [8]. In this concern, metal nanoparticles like zinc, silver, and titanium could enhance the gas barrier ability of biodegradable polymer films [9,10]. The silver nanoparticles (AgNPs) have been used to prepare nanocomposites in food packaging applications because of their high thermal stability. For example, Shankar et al. (2018) found that the permeation ability of water vapour significantly decreased by adding lignin and Ag nanoparticles [11,12]. This trend may be due to changes in the interaction between molecules that cause the path of the water vapor molecules diffusing through the polymer matrix to become more tortuous. In our previous work, we tried to prepare PLA/AgNPs nanocomposites with various AgNPs loading (0 wt.%, 1 wt.%, 3 wt.%, 5 wt.%, 10 wt.%, and 15 wt.%). Oxygen transmission rate (OTR) value for PLA nanocomposites with 3 wt.% AgNPs was found to be the lowest among all the samples. The PLA nanocomposite film with 5 wt.% AgNPs loading had the lowest water vapour permeability (WVP) value (data not shown in this article). So, PLA/AgNPs nanocomposites with 3 wt.% or 5 wt.% AgNPs loading were selected for use in this work.

High pressure treatment is a new technology in food preservation and food processing which works by regulating pressure at normal or low temperature. However, in an attempt to improve the physicochemical properties of the film, high pressure processing of the PLA-based formulation has been used to prepare the film. The film-forming liquid is treated with high pressure, which is a novel technology that has been only reported in recent years [13,14,15]. For the majority of films studied, the literature has reported an increase in water vapor or oxygen barrier properties, along with a decrease in the migration of substances from polymer composites. Kang & Min (2010) reported that potato peel-based film developed using high pressure treatment (138 MPa), ultrasound technology and irradiation [16]. High pressure homogenization could enhance the water vapour barrier and mechanical properties of films when compared with those treated by ultrasound or irradiation.

As far as we know, there is still no detailed study on the effect of high pressure treatment on nanoparticles dispersion in film-forming solutions and physiochemical properties of resultant nanocomposite films. In this context, this experiment was to investigate the effect of high pressure treatment on PLA/AgNPs nanocomposite film-forming solution to modify the microstructure of films by determining its impact on the structure of macromolecules. The resultant films were also studied with respect to water vapour barrier, thermal, and mechanical properties. Additionally, the migration behavior of AgNPs from the PLA nanocomposites to food simulants was determined by inductively coupled plasma atomic emission spectroscopy (ICP-AES).

## 2. Material and Methods

### 2.1. Materials

Commercially available poly(l-lactic acid) (PLA) (280 kDa of molecular weight) was obtained from Nature Works LLC (Blair, NE, USA). Silver nanoparticles (AgNPs) were purchased from Wanjing New Material Co., Ltd. (Hangzhou, China), with an average particle size < 150 nm and purity of 99.0%. Tributyl acetocitrate (ATBC), a plasticizer, and chloroform, a solvent, were supplied by Chengdu Kelong Chemical Co., Ltd. (Sichuan, China). The reagents used in this work were of analytical grade.

### 2.2. Preparation of PLA/AgNPs Composite Films

PLA/AgNPs composite film was prepared by a solvent evaporation method. PLA film was prepared as control. PLA-plasticizer dispersions were prepared by dissolving 4 g of pre-dried PLA and 0.4 g of ATBC in 50 mL of chloroform by stirring 6 h with a magnetic agitator. AgNPs at 3 wt.% or 5 wt.% was incorporated into the PLA film-forming solution and stirred for 2 h. Then, the solution with AgNPs was ultrasonically degassed to remove air bubbles. PLA/AgNPs composite film incorporated with 3 wt.% and 5 wt.% AgNPs was named as PLA/AgNPs-3 and PLA/AgNPs-5, respectively.

High pressure equipment was used to treat the film-forming solution directly and resulted in significant effects on the microstructure of polymer solution. In this work, 200 MPa and 400 MPa were selected to treat the PLA/AgNPs film-forming solution. The solution was placed in a vacuum bag and high pressure treatment was performed (Freshertech-Hpp, BaoTou Kefa High Pressure Technology Co., Ltd., Nei Menggu, China) at 0, 200 and 400 MPa for 15 min at room temperature, using water as the pressure transmitting fluid. After the solution was treated with high pressure, it was evenly poured into a glass plate (20 cm × 20 cm), the glass plate was placed in a dry environment, to be completely volatilized, and a nanocomposite film was formed at room temperature. After drying, the PLA/AgNPs composite film was peeled and kept in a desiccator at 23 ± 2 °C and relative humidity of 50 ± 5% for at least 48 h prior to characterization. Thickness was measured by a digital micrometer (Mitotuyo 7327, Tokyo, Japan). The thickness of PLA, PLA/AgNPs-3, PLA/AgNPs-5, PLA/AgNPs-3/200MPa, PLA/AgNPs-3/400MPa, PLA/AgNPs-5/200MPa and PLA/AgNPs-5/400MPa film was 45 μm, 49 μm, 51 μm, 48 μm, 48 μm, 49 μm and 50 μm, respectively.

### 2.3. Microstructure

The microstructure of PLA/AgNPs composite film was investigated using field emission scanning electron microscopy (FE-SEM) (S-4800, Hitachi Co., Ltd., Tokyo, Japan) with an accelerating voltage of 5 kV. To examine the cross-section morphology of samples, the film was immersed in liquid nitrogen and fractured by tweezers.

### 2.4. Water Vapour Permeability (WVP) Measurement

WVP of the PLA/AgNPs composite film was measured gravimetrically according to ASTM E-96 standard method with some modifications [6]. 12 g anhydrous silica gel was placed in the weighing bottle, the test films were tightly covered and fixed on the weighing bottle with paraffin and rubber, weighed, and the weighing bottle placed in the bottom of a desiccator filled with saturated NaCl. Finally, the desiccator was placed in an environment at a temperature of 25 °C and a relative humidity of 65% for 24 h and weighed at a given time. Results were based on 3 replications.

The water vapor transmission rate is calculated according to the following formula:

(1) $$WVP=\frac{\Delta w\times X}{\Delta t\times A\times \Delta P}\;$$

Where Δ*w* is the change in weight of the weighing bottle (g), *X* is the average film thickness (m), Δ*t* is the measured time (s), *A* is the area of the test area (m^2^), Δ*P* is the difference in water vapor pressure across the films.

### 2.5. Thermal Analysis

Thermal analysis of the PLA/AgNPs composites was performed using differential scanning calorimetry (DSC) (214, Netzsch, Selb, Germany). Samples around 10 mg were weighed and hermetically sealed in an aluminum pan. The film sample was heated from 20 to 200 °C at a rate of 10 °C/min in a nitrogen atmosphere and then cooled from 200 to 20 °C at a rate of 10 °C/min. Finally, the temperature was further raised to 200 °C at 10 °C/min. The first heating scan was used to eliminate any prior thermal history of the sample. An empty pan was used as the reference. The crystallization temperature (*T_c_*), melting temperature (*T_m_*), and glass transition temperature (*T_g_*) were obtained from the second heating/cooling cycle of DSC curves. 3 replications of each sample were tested. In addition, the percentage of crystallinity (*X_c_*) was calculated according to Equation (2) [17]:

(2) $$X_{c}(\%)=(\Delta H_{m}/\Delta H_{m}^{0}w)\times 100\;$$

Where Δ*H_m_* is the enthalpy of melting (J/g), $\Delta H_{m}^{0}$ is the heat of fusion for completely crystalline PLA (93.7 J/g), and *w* is the weight fraction of PLA in the samples.

### 2.6. Mechanical Property

Tensile strength (*σ*), percentage elongation at break (*ε*), and elastic modulus (*E*) of films were evaluated from stress-strain curves obtained by tensile testing equipment (CMT 4104, MTS Systems Co., Ltd., Guangzhou, China). Five specimens were cut from each of the PLA/AgNPs composite films with a size of 15 mm × 100 mm. The cross-head speed was set at 50 mm/min.

### 2.7. Migration Test

The AgNPs migration test was performed at room temperature. 50% (*v/v*) ethanol was used as alcoholic food simulant to investigate the migration of AgNPs from the PLA film. A square film sample (40 mm × 40 mm) was immersed in a glass tube with 25 mL of food simulant as indicated in the legislation (European Food Safety Agency). Samples in 50% (*v/v*) ethanol were kept at 40 °C for 40 days [18]. At 0, 5, 10, 15, 20, 30 and 40 days of storage, films were removed and the amount of AgNPs in the simulant solution was analyzed by an inductively coupled plasma atomic emission spectrophotometer (ICP-AES, Optima 8000, Perkin Elmer, Waltham, MA, USA). The migration test was carried out in triplicate and the overall migration value was expressed as μg/kg.

### 2.8. Statistical Analysis

The tests were determined by an analysis of variance (ANOVA) using a Statistical Product and Service Solutions (SPSS) system, version 13.0. The mean comparison was performed by the Duncan’s multiple range tests. The significance level was set at *p* < 0.05.

## 3. Results and Discussion

### 3.1. Microstructure

**Figure 1:**
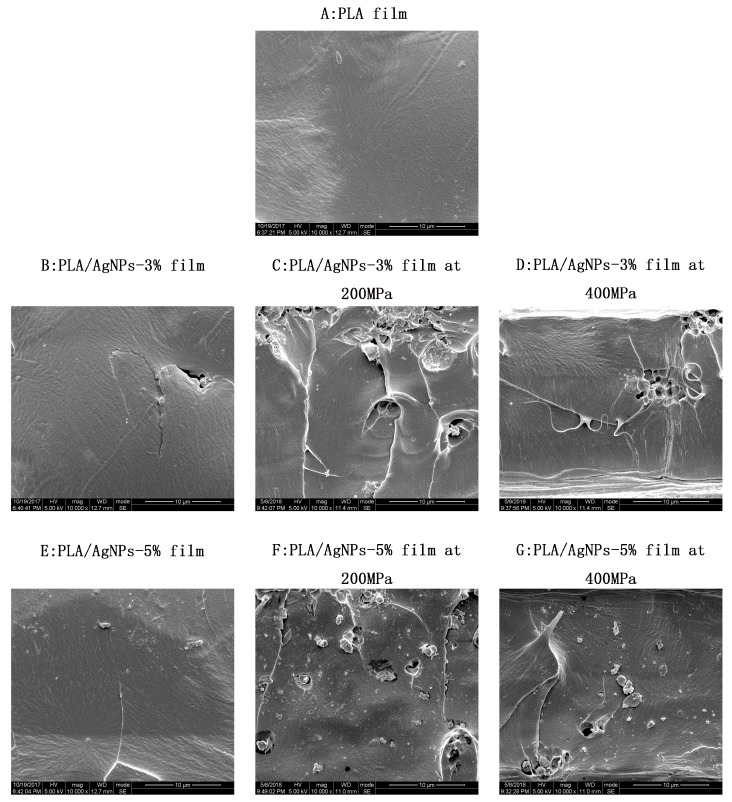
Effect of high pressure treatment on the microstructure of poly (lactic acid) (PLA)/silver nanoparticles (AgNPs) nanocomposite films (magnification: 10,000×): (**A**) PLA film; (**B**–**D**) PLA/AgNPs-3 film treated at 0, 200 and 400 MPa, respectively; (**E**–**G**) PLA/AgNPs-5 film treated at 0, 200 and 400 MPa, respectively.

Figure 1 shows the microstructure of PLA/AgNPs nanocomposite films. As in Figure 1A, the PLA film without AgNPs loading and high-pressure treatment was smooth. Figure 1B,E depicts the cross-section images of PLA/AgNPs-3 and PLA/AgNPs-5 films. After AgNPs were incorporated into the polymer matrix, a few pits on the cross-section surface appeared. This might be because the nanoparticle was not dissolved in polymer solution. However, the cross-section surface was rougher for the films treated by high pressure compared to those without high pressure treatment. The interactions between PLA and plasticizer were promoted by high pressure treatment [19]. Shankar et al. reported that the roughness of the PLA/ZnO NPs composite film increased with the increase in the content of ZnO NPs and the WVP of the nanocomposite film had decreased when ZnO NPs were used as reinforcing nanofiller [20]. Through SEM analysis on biopolymer films, Monteiro et al. also indicated that a more uniform surface without the presence of agglomerate along the polymer matrix would bring fragility fragment susceptible to water vapour diffusion to the biopolymer films [21]. When the high pressure is increased from 200 to 400 MPa, it can be observed that the cross section becomes flatter and the pits become less. A similar trend can be seen in the reports by Lian et al. (2016), that a smooth appearance for nanocomposite films treated at 600 MPa was observed [15].

### 3.2. WVP of PLA and Its AgNPs Nanocomposite Films

Effect of high pressure treatment on the WVP of PLA/AgNPs nanocomposite films was calculated in Table 1. The WVP of PLA film (5.8 ± 0.1 × 10^−10^ g·m/m^2.^Pa·s) was higher than that reported in our previous work (1.1 ± 0.2 × 10^−10^ g·m/m^2.^Pa·s) [6]. This could be because of the addition of 9 wt.% ATBC, a highly hydrophilic plasticizer. Gao et al. (2016) also reported that the WVP value increased as tributyl citrate concentration increased from 0 to 10% [22]. The incorporation of plasticizer into the polymer matrix could improve the flexibility of films, by leading to a decline in the gas barrier property.

Some factors could affect the gas barrier property of polymer films, including the crystalline, integrity, and chain mobility [15]. Reductions in WVP values with respect to PLA films when adding AgNPs were shown in nanocomposite films. PLA/AgNPs-3 and PLA/AgNPs-5 showed a reduction of 7.8% and 26.1%, respectively. This might be because of the tortuous way it was induced by the impervious nanoparticles in the polymer matrix, and because the path of water vapour diffusion was prolonged and as the nanoparticles are added, the crystallization is increased, and the curvature of the water vapor transport path is further improved [23,24].

High pressure treatment at 200 MPa or 400 MPa significantly (*p* < 0.05) improved the barrier property of PLA/AgNPs nanocomposite films with 3 wt.% and 5 wt.% AgNPs loading. High pressure treatment increased the interaction between PLA and nanoparticles according to the SEM analysis, thus decreasing the WVP value of PLA/AgNPs nanocomposite films. PLA/AgNPs-5 group treated at 200 MPa had the lowest WVP value when compared to other groups. In all of the samples, high pressure treatment at 200 MPa could effectively lower the WVP values by 32.9%, 41.2%, and 51.5% for PLA, PLA/AgNPs-3, and PLA/AgNPs-5, respectively. The WVP value for PLA, PLA/AgNPs-3, and PLA/AgNPs-5 films increased when the pressure increased up to 400 MPa, whereas it was still significantly (*p* < 0.05) lower than that of films without high pressure treatment. This might be due to the smooth and flat images confirmed by SEM analysis, indicating that PLA/AgNPs nanocomposite films treated at 400 MPa were homogenous, thus it increased the WVP value in 400 MPa treated films.

AgNPs could fill in the polymer network structure. In Table 2, it is known that high pressure treatment improves the crystallinity of the composite film and makes the internal arrangement of the composite film more compact, so its network structure is compressed and the permeability of water vapor is reduced [15,25]. Aulin et al. (2010) also reported that intermolecular interactions, such as hydrogen bonds and van der Waals force, could induce a more compact network and lower free volume associated with higher gas barrier property [26]. The results showed that AgNPs incorporation and high pressure treatment could effectively improve the gas barrier property of PLA/AgNPs nanocomposite films, and high pressure treatment was preferred.

### 3.3. Thermal Analysis

**Figure 2:**
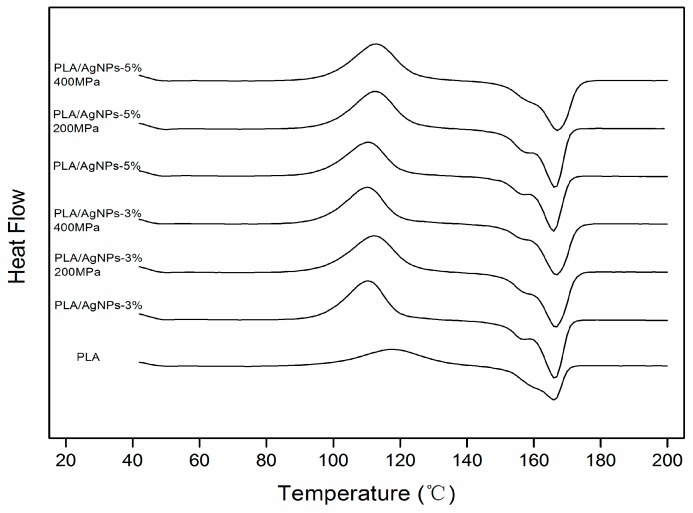
Effect of high pressure treatment on the differential scanning calorimetry (DSC) thermograms of PLA/AgNPs nanocomposite films (PLA film; PLA/AgNPs-3 film treated at 0, 200 and 400 MPa; PLA/AgNPs-5 film treated at 0, 200 and 400 MPa, respectively).

DSC was performed to analyze the thermal transitions of PLA nanocomposites. The temperature (*T_g_*, *T_c_* and *T_m_*) of the different thermal events during the second heating run were listed in Table 2 and Figure 2. The combination of AgNPs to PLA matrix did not reveal significant differences with respect to PLA in regard to *T_g_* and *T_m_*, in agreement with previous studies [27]. High pressure treatment on film-forming solution only significantly affected *T_g_*, *T_c_*, and *T_m_* of PLA nanocomposites. *T_c_* value of PLA/AgNPs-5 nanocomposites slightly increased from 110.4 to 112.9 °C in parallel with the increase in *T_g_*, when the high pressure increased from 0 to 400 MPa. *T_c_* value of PLA/AgNPs-5 nanocomposites slightly increased in parallel with the increase in *T_g_* value. This indicated that high pressure treatment would decrease molecular chain mobility of the PLA chains [28]. It can be found from Table 2 that the crystallinity of the composite film after high pressure treatment is significantly improved. Yoo et al. (2010) also found that the crystallinity of polymers was affected by temperature and pressure, and it increases with increasing temperature and pressure [25].

### 3.4. Mechanical Property

Effects of high pressure treatment on the mechanical property of PLA/AgNPs nanocomposite films were listed in Table 3. Tensile strength (*σ*) value for PLA, PLA/AgNPs-3, and PLA/AgNPs-5 films without high pressure treatment was 29.9 MPa, 32.9 MPa and 33.8 MPa, respectively. Adding nanoparticles turns out to be effective in improving the stiffness and strength of a given polymer [29]. However, adding AgNPs in higher content, 5 wt.%, could not significantly (*p* > 0.05) increase the *σ* value of PLA/AgNPs nanocomposite films. High pressure treatment at 200 MPa or 400 MPa significantly (*p* < 0.05) affected the mechanical property of PLA/AgNPs-3 and PLA/AgNPs-5 films. When the high pressure increased from 0 to 400 MPa, σ value for PLA/AgNPs-3 film increased from 33 to 36 MPa, and *σ* value for PLA/AgNPs-5 film increased from 34 to 36 MPa. The mechanical force applied during high pressure treatment increased the stiffness of nanocomposite films through the development of hydrogen bonds [14]. Similar findings were also reported by Fu et al. (2011), by applying high pressure homogenization on the film-forming solution based on starch and plasticizer, and by Molinaro et al. (2015), by applying high pressure treatment on a gelatin-based film-forming solution [13,14]. High pressure treatment could reduce the aggregation of nanoparticles and make them fully dispersing in film-casting solution. High pressure treatment was a physical method to promote the formation of denser three-dimensional networks between nanoparticles and polymer matrix, thus increasing *σ* value of nanocomposite polymer films [15].

*E* value for PLA, PLA/AgNPs-3, and PLA/AgNPs-5 films without high pressure treatment was 1083 MPa, 1123 MPa and 1142 MPa, respectively. Percentage elongation at break (*ε*) value for PLA, PLA/AgNPs-3 and PLA/AgNPs-5 films without high pressure treatment was 194%, 179% and 170%, respectively. The addition of AgNPs in higher content produced PLA nanocomposite films with lower elongation property. Nanoparticles could improve the strength of films at the expense of a reduction in elongation at break [30].

*E* value for PLA/AgNPs-3 film treated by high pressure originally increased to 1664 MPa and then increased to 1930 MPa. Meanwhile, *E* value for PLA/AgNPs-5 film treated by high pressure originally increased to 1369 MPa and then increased to 1470 MPa. A similar trend was also found in *σ* value of PLA/AgNPs nanocomposite films treated by high pressure. This was because polymer nanocomposite films treated by high pressure had higher crystallinity. The elongation property decreased with the increase in the crystallinity of films [13]. High pressure treatment could induce a more compact structure and the films gained from the homogenized dispersion were more fragile than those gained from non-homogenized film-forming solution.

### 3.5. Migration Test

The migration test was carried out according to European Food Safety Agency (EFSA), which is a procedure to evaluate the migration of additives in food packaging materials coming into contact with foodstuffs [31,32]. Migration behavior of nanoparticles is one of the most important factors to assess the applicability of PLA nanocomposite films [33].

The total amount of AgNPs that migrated from PLA/AgNPs nanocomposite films into a simulant solution under different high pressure was listed in Table 4. With the increase of storage time of all samples, the amount of AgNPs migration increased. The amount of AgNPs which migrated from the PLA/AgNPs-5 film was significantly (*p* < 0.05) higher than in the PLA/AgNPs-3 group. This trend was also reported by Fortunati et al. (2012), who researched the release of silver nanoparticles from PLA nanocomposites to foodstuff [34]. The migration amount sharply increased before 20 days of storage and then gradually increased till the end of storage. Adding nanoparticles to the PLA matrix accelerates the rate of hydrolysis degradation and thus increased the additive migration [35]. Fortunati et al. (2013) proposed that AgNPs migration mechanism was response to the plastic of packaging films [9]. AgNPs could improve the strength of films at the expense of a reduction in elasticity. This was confirmed by the mechanical test.

The migration amount for films treated by 200 MPa or 400 MPa was significantly (*p* < 0.05) lower than that for films without high pressure treatment. Furthermore, the films treated by 200 MPa exhibited the lowest migration level. At 40 days of storage, the maximum migration amount was observed in the PLA/AgNPs-5 film without high pressure treatment, and the minimum migration amount was found in the PLA/AgNPs-3 film treated by 200 MPa. This might be because high pressure treatment could make the polymer network much denser. The strong interactions between nanoparticles and polymer matrix would decrease the chain mobility and prevent oxygen and moisture uptake [36]. Xiang et al. (2013) also reported that a denser morphology in nanocomposites would produce a slower release rate of nanoparticles from PLA matrix [35]. This trend was consistent with the mechanical properties of nanocomposite films treated by high pressure.

Although the AgNPs migration amount of PLA/AgNPs-5 film treated by 200 MPa and 400 MPa was 354 μg /kg and 409 μg /kg, respectively, it was still far less restrictive than EFSA’s restriction on 10 mg/kg for food contact materials. Furthermore, the AgNPs migration amount in this study was lower than that from polyethylene-Ag nanocomposite film or PLA-cellulose-Ag nanocomposite film without high pressure treatment [9,37]. The AgNPs migration amount from PLA nanocomposites treated by high pressure was extremely little when compared with that incorporated, and it was considered safe. The PLA/AgNPs nanocomposite film in this study was suitable for contact with foodstuffs.

## 4. Conclusions

To improve the water vapour barrier property of PLA/AgNPs nanocomposite film, high pressure (200 MPa or 400 MPa) was applied to the film-forming solution. We found that the effect of PLA/AgNPs-5 film treated by 200 MPa is the best. SEM morphology revealed that high pressure treatment could provide a more tortuous way for water vapour or oxygen to transfer through PLA film. Thermal analysis indicated that high pressure decreased molecular chain mobility of the PLA chains. High pressure treatment leads to an increase in crystallinity of the polymer, which makes the structure of the nanocomposite film more compact. Mechanical performance analysis shows that high pressure treatment can enhance the stiffness of the nanocomposite film through the development of hydrogen bonds, and decrease the elongation of the nanocomposite film. The AgNPs migration amount from PLA nanocomposites treated by high pressure was extremely little. This finding was significant for developing a safe food packaging material with higher water vapour barrier properties.

## Figures and Tables

**Table 1:** Effect of high pressure treatment on the water vapour permeability of PLA/AgNPs nanocomposite films.

Samples AgNPs (wt.%)	Pressure (MPa)	WVP × 10^−10^ (g·m/m^2^·s·Pa)	Reduction in WVP (%)
0	0	5.8 ± 0.1 ^a^	-
3	0	5.3 ± 0.2 ^b^	7.8
3	200	3.4 ± 0.2 ^de^	41.2
3	400	3.6 ± 0.1 ^d^	37.9
5	0	4.3 ± 0.3 ^c^	26.1
5	200	2.8 ± 0.1 ^f^	51.5
5	400	3.2 ± 0.2 ^e^	44.3

^a–f^ Values followed by different superscripts in the same column denote significant difference (*p* < 0.05), where a is the highest value.

**Table 2:** Effect of high pressure treatment on the mechanical property of PLA/AgNPs nanocomposite films.

SamplesAgNPs (wt.%)	Pressure (MPa)	*T_g_* (°C)	*T_c_* (°C)	*T_m_* (°C)	*Xc* (%)
0	0	49.9 ± 0.3 ^c^	116.6 ± 0.2 ^a^	166.7 ± 0.4 ^a^	12.9 ± 0.3 ^g^
3	0	50.5 ± 0.4 ^bc^	112.7 ± 0.8 ^b^	166.3 ± 0.4 ^a^	14.3 ± 0.4 ^f^
3	200	50.5 ± 0.1 ^bc^	112.2 ± 0.3 ^b^	166.5 ± 0.2 ^a^	25.5 ± 0.5 ^b^
3	400	50.3 ± 0.1 ^bc^	110.2 ± 0.2 ^c^	166.8 ± 0.1 ^a^	28.7 ± 0.7 ^a^
5	0	50.1 ± 0.2 ^c^	110.4 ± 0.4 ^c^	165.9 ± 0.2 ^a^	15.8 ± 0.6 ^e^
5	200	50.9 ± 0.5 ^ab^	112.4 ± 0.6 ^b^	166.5 ± 0.8 ^a^	20.5 ± 0.6 ^d^
5	400	51.9 ± 0.2 ^a^	112.9 ± 0.5 ^b^	167.0 ± 0.3 ^a^	23.9 ± 0.4 ^c^

^a–g^ Values followed by different superscripts in the same column denote significant difference (*p* < 0.05), where a is the highest value.

**Table 3:** Effects of high pressure treatment on the mechanical property of PLA/AgNPs nanocomposite films.

Samples AgNPs (wt.%)	Pressure (MPa)	*σ* (MPa)	*E* (MPa)	*ε* (%)
0	0	30 ± 3 ^d^	1083 ± 89 ^d^	194 ± 6 ^a^
3	0	33 ± 2 ^bc^	1123 ± 74 ^d^	179 ± 13 ^b^
3	200	34 ± 2 ^ab^	1664 ± 101 ^b^	159 ± 10 ^c^
3	400	36 ± 2 ^a^	1930 ± 70 ^a^	129 ± 9 ^d^
5	0	34 ± 2 ^ab^	1142 ± 95 ^d^	170 ± 8 ^b^
5	200	35 ± 2 ^ab^	1369 ± 62 ^c^	161 ± 14 ^c^
5	400	36 ± 2 ^a^	1479 ± 89 ^c^	119 ± 14 ^d^

^a–d^ Values followed by different superscripts in the same column denote significant difference (*p* < 0.05), where a is the highest value.

**Table 4:** Effect of high pressure treatment on the migration behavior of PLA/AgNPs nanocomposite films.

Samples AgNPs (wt.%)	Pressure (MPa)	5d (μg/kg)	10d (μg/kg)	20d (μg/kg)	30d (μg/kg)	40d (μg/kg)
3	0	138 ± 17 ^aBC^	223 ± 15 ^bAB^	348 ± 16 ^cABC^	357 ± 25 ^cBC^	369 ± 20 ^cAB^
3	200	82 ± 9 ^aA^	181 ± 11 ^bA^	302 ± 27 ^cA^	288 ± 14 ^cA^	312 ± 18 ^cA^
3	400	99 ± 11 ^aAB^	182 ± 17 ^bA^	330 ± 25 ^cAB^	339 ± 11 ^cAC^	341 ± 21 ^cAB^
5	0	169 ± 12 ^aC^	262 ± 22 ^bB^	412 ± 17 ^cC^	424 ± 18 ^cD^	434 ± 13 ^cC^
5	200	128 ± 23 ^aABC^	229 ± 13 ^bAB^	333 ± 13 ^cAB^	341 ± 16 ^cCB^	354 ± 10 ^cAB^
5	400	131 ± 12 ^aABC^	235 ± 18 ^bAB^	385 ± 15 ^cBC^	395 ± 18 ^cAD^	409 ± 16 ^cAC^

^a–c^ Values followed by different superscripts in the same row denote significant difference (*p* < 0.05), where a is the lowest value; ^A–D^ Values followed by different superscripts in the same column denote significant difference (*p* < 0.05), where A is the lowest value.
